# Severe mpox (formerly monkeypox) disease in five patients after recent vaccination with MVA-BN vaccine, Belgium, July to October 2022

**DOI:** 10.2807/1560-7917.ES.2022.27.48.2200894

**Published:** 2022-12-01

**Authors:** Nicole Berens-Riha, Tessa De Block, Jojanneke Rutgers, Johan Michiels, Liesbeth Van Gestel, Matilde Hens, ITM monkeypox study group, Chris Kenyon, Emmanuel Bottieau, Patrick Soentjens, Johan van Griensven, Isabel Brosius, Kevin K Ariën, Marjan Van Esbroeck, Antonio Mauro Rezende, Koen Vercauteren, Laurens Liesenborghs, ITM Monkeypox Study Group, Christophe Van Dijck, Irith De Baetselier, Dorien Van den Bossche, Eric Florence, Wim Adriaensen, Jasmine Coppens, Fien Vanroye, Kadrie Ramadan, Karin Van Looveren, Jolien Baeyens, Leo Heyndrickx, Hanne Rasson, Jacob Verschueren, Stefanie Bracke, Leen Vandenhove, Jef Vanhamel, Bea Vuylsteke

**Affiliations:** 1 Institute of Tropical Medicine, Antwerp, Belgium; 2 Additional members of the study group are listed under Collaborators.; 3 University of Cape Town, Cape Town, South Africa

**Keywords:** monkeypox, mpox, MPX vaccination, proctitis, penile oedema, severe MPX, off-label vaccination

## Abstract

Vaccination is important in containing the 2022 mpox (formerly monkeypox) epidemic. We describe five Belgian patients with localised severe symptoms of proctitis and penile oedema, occurring between 4 and 35 days after post-exposure preventive vaccination or after one- or two-dose off-label pre-exposure preventive vaccination with MVA-BN vaccine. Genome sequencing did not reveal evidence for immune escape variants. Healthcare workers and those at risk should be aware of possible infections occurring shortly after vaccination and the need for other preventive measures.

By 22 November 2022, 20,887 mpox (formerly monkeypox) cases have been reported in Europe [1]. However, since August 2022, the incidence has steeply declined, which could be a result of several factors including behavioural change and an increasing level of immunity within the population at risk, either naturally acquired or through targeted vaccination campaigns. Because of a vaccine shortage, several countries including Belgium introduced off-label vaccination regimens. Here, we report a case series of five Belgian patients presenting with severe mpox disease shortly after post-exposure preventive vaccination (PEPV), or after one- or two-dose off-label primary preventive vaccination (PPV) in non-primed individuals.

## Off-label mpox vaccination in Belgium

In Belgium, mpox vaccination with the modified vaccinia Ankara (MVA-BN; Bavarian Nordic) vaccine started with PEPV end of May 2022. From the end of July, the main Belgian sexually transmitted infection (STI) clinics began PPV campaigns with administration of a first subcutaneous (SC) dose. In absence of immunosuppression, the second dose was delayed, instead of being given at the recommended interval of 28 days. In agreement with recommendations from several health councils, vaccinations from 2 September onwards were exclusively given off-label via the intradermal (ID) route, at one fifth of the SC dose. Since the end of November, the vaccine supply was secured and was again given as two SC doses with an interval of 28 days [2].

By 28 November 2022, 1,408 individuals had been vaccinated at our institute with a first dose and 909 with a second dose, mostly ID. The PPV vaccination from July to August was restricted to MSM with at least one STI in the last year, to male and transgender sex workers, immunocompromised MSM and laboratory personnel working with viral culture; since September all MSM gradually became eligible. No follow-up of the vaccinees was established. However, vaccinees were advised to contact us in case of any suspicious symptoms. Three patients presented with symptoms compatible with mpox after vaccination at our institute between the end of July and beginning of October, and two others were diagnosed with mpox at an emergency department and contacted us after the diagnosis in October.

## Confirmed symptomatic mpox cases shortly after vaccination

**Figure 1:**
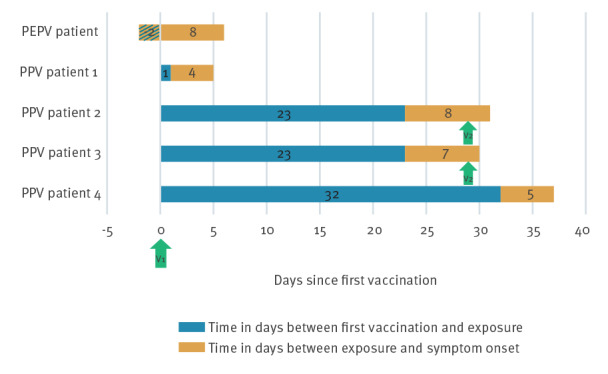
Timeline of vaccination, exposure and symptom onset for five mpox patients, Belgium, July–October 2022

PEPV: post-exposure preventive vaccination; PPV: primary preventive vaccination; V1: vaccination 1; V2: vaccination 2.

The Table describes relevant demographic, behavioural, laboratory and clinical data of five patients with PCR-confirmed monkeypox virus (MPXV) infection after vaccination. All patients identified as cis MSM. Their median age was 38 years (range: 34–47). Two patients were HIV-positive under effective anti-viral treatment (ART). All five patients received at least one full dose SC of the MVA-BN vaccine, four as PPV and one as PEPV. Two patients were given a second preventive vaccination dose ID, 29 days after the first dose. Figure 1 gives an overview of the five patients and the timing of infection in relation to the vaccines received. Time from the first and second dose to symptom onset ranged from 4 to 35 days and 1 to 2 days, respectively. The exposure was reported between 2 days before (PEPV patient) and 32 days after vaccination.

**Table:** Demographic, laboratory and clinical data and information on vaccination and exposure from five mpox patients, Belgium, July–October 2022

Characteristics	Mpox patients				
	PEPV patient	PPV patient 1	PPV patient 2	PPV patient 3	PPV patient 4
Baseline data					
Gender	Cis man	Cis man	Cis man	Cis man	Cis man
Age range (years)	31–40	31–40	31–40	41–50	41–50
Health status					
HIV status	Negative	Positive	Negative	Positive	Negative
CD4^+^ T-cell count (cells/µl)	NA	1,110	NA	1,056	NA
Viral load (copies/ml)	NA	22	NA	Not detectable	NA
HIV PrEP	Yes	No	Yes	No	Yes
Immunosuppression	No	Yes (immune therapy, malignancy)	No	No	No
Vaccination status					
Indication of recent vaccine	PEPV	PPV	PPV	PPV	PPV
Route of first vaccination	Subcutaneous	Subcutaneous	Subcutaneous	Subcutaneous	Subcutaneous
Route of second vaccination	NA	NA	Intradermal	Intradermal	NA
Previous smallpox vaccination	No	No	No	Unknown, no scar	No
Risk group/exposure					
Health worker	No	No	No	No	No
Mass event	No	No	No	No	No
Sexual orientation	MSM	MSM	MSM	MSM	MSM
Sexual preference^a^	Bottom, oral, petting	Top, oral	Bottom, top	Bottom, top	Bottom, oral
Number of sexual partners at exposure^b^	1	1	3	3	1
Condom use	No	No	No	No	No
PCR Cq^c^ values (day of symptoms)					
Anorectal swab	17.3 (day 1)	19.7 (day 3)	25.1 (day 12)	35.8 (day 13)	19.8 (day 16)
Saliva	36.8 (day 2)	NA	Negative	NA	29.8 (day 16)
Throat swab	Negative	Negative	NA	NA	NA
Skin swab	NA	NA	NA	NA	26.1 (day 16)
Blood	35.3 (day 4)	NA	NA	NA	36.0 (day 16)
Genital swab	37.8 (day 1)	34.3 (day 3)^d^	NA	NA	20.4 (day 16)
Viral culture (swab origin)	Positive (anal)	Positive (anal)	Positive (anal)	Not done^e^	Positive (anal, genital)
Clinical data					
Prodromal symptoms	Yes	Yes	Yes	Yes	Yes (prolonged)
Fever	Yes	Yes	None	None	Yes
Skin lesions, location (n)	None	None	None	None	Face (4)
Mucosal lesions, location (n)	Anal (5–25)	Glans penis (5–25)	Anal (5–25)	Anal (5–25)	Anal/peri-anal (0–4)
Severity^f^	Severe	Severe	Severe	Severe	Severe
Complications	Proctitis (rectal pain, mucus, pus, blood)	Penile oedema with bacterial superinfection, circumcision performed	Proctitis (rectal pain, mucus, pus)	Proctitis (rectal pain, mucus, blood, pus)	Proctitis (rectal pain, diarrhoea) dysphagia, mild super-infection of facial lesions
Treatment	Ceftriaxone,doxycycline, mesalazine, prednisolone, paracetamol, tramadol	Clindamycin, paracetamol	Ceftriaxone, azithromycin, paracetamol	Ceftriaxone, azithromycin, paracetamol	Fucidine (topical), Xylocaine (topical, 5%), paracetamol

Cq; quantification cycle; HIV: human immundeficiency virus; MSM: men who have sex with men; NA: not applicable; PEPV: post-exposure preventive vaccination; PPV: primary preventive vaccination; PrEP: pre-exposure prophylaxis.

^a^ ‘Bottom’ refers to receptive anal sex and ’top‘ refers to insertive anal sex.

^b^ PPV patients 1 and 4 had sex with unknown contacts, the PEPV patient and PPV patients 2 and 3 had sex with confirmed mpox cases (confirmed after exposure according to information from patients).

^c^ Cq values from mpox real-time PCR. Low values indicate higher viral load of the sample. The day of first positivity after symptom onset is listed in parentheses. Methods listed in [3].

^d^ Sample was urine.

^e^ Cq value was regarded as too high to attempt viral culture.

^f^ According to our in-house severity scale for outpatient clinic patients, defined in Supplementary Figure S1, all were graded as severe.

All five patients developed localised anogenital symptoms, matching with the presumed location of exposure (Table 1). Additional clinical details are described in Supplementary Table S1. All three patients who received only a single vaccine dose presented with fever and other systemic symptoms, including fatigue, headache and/or cough, while the two double vaccinated patients did not report fever. According to our outpatient clinic severity scale defined in Supplementary Figure S1, all patients suffered from severe symptoms. Four patients presented with proctitis requiring antibiotics and analgesics including opioids. One of these patients additionally developed large partly necrotic facial ulcerations, requiring antibiotics and local anaesthesia. The fifth patient developed penile oedema with bacterial superinfection for which circumcision was needed. Symptoms in the two patients who received a second dose ID were less severe compared with the others, i.e. the lesions were less painful and symptoms resolved faster. Nevertheless, both presented at an external emergency department in need of antibiotic and pain treatment.

Diagnosis of mpox by quantitative PCR (done according to [3]) was performed between day 1 and 16 after symptom onset. Anal swabs from the three patients who received one dose showed cycle quantification (Cq) values below 20. In PPV patient 4, who reported prolonged fever and shivering, viral DNA could still be detected in blood samples at day 16. The double vaccinated patients presented with higher Cq values, although anal swabs were only taken on day 12 and 13 after symptoms were resolved (Table 1 and Supplementary Table S1). Viral culture [3] confirmed the presence of replication-competent MPXV in different swabs from four of the five patients.

## Whole genome sequencing

**Figure 2:**
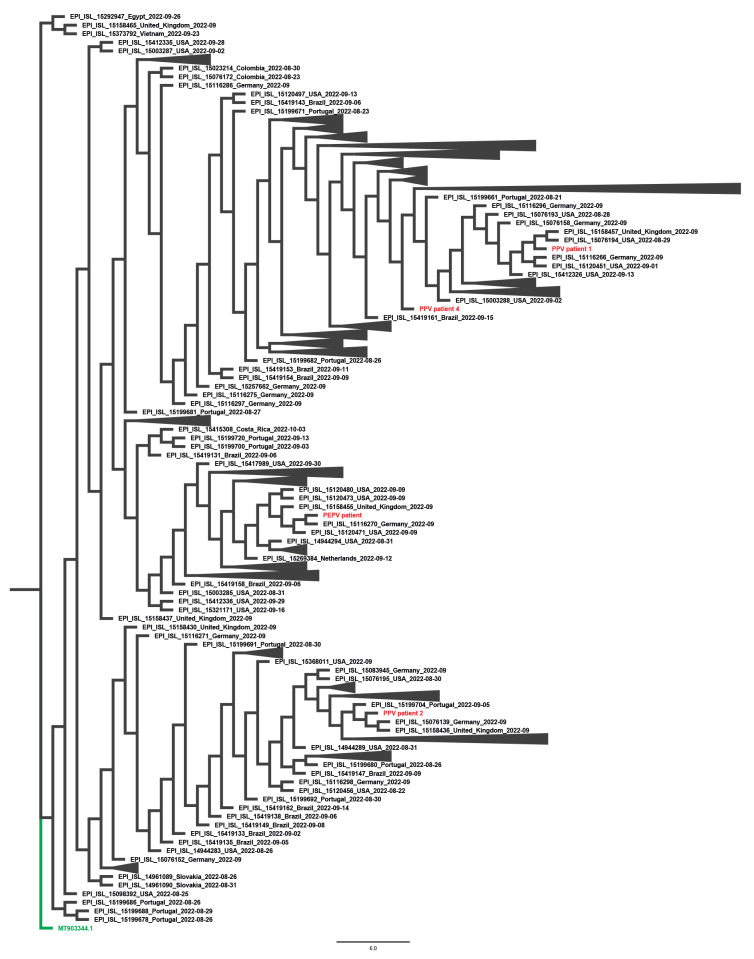
Phylogeny of the virus genome of four mpox patients, July–October 2022, compared to 479 monkeypox virus Clade IIb sequences available on GISAID from 21 August–21 October 2022, Belgium

PEPV: post-exposure preventive vaccination; PPV: primary preventive vaccination.

**Figure 3:**
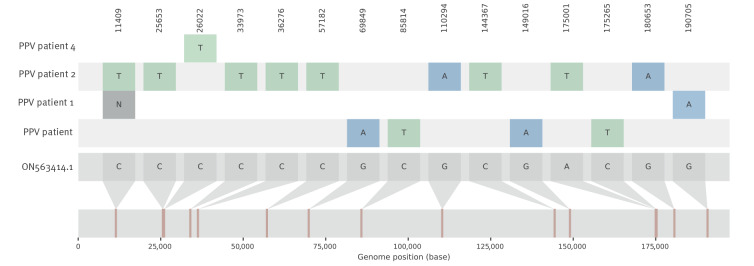
Single nucleotide variant analysis of the virus genome of four mpox patients compared with a 2022 reference genome from the United States, Belgium, July–October 2022

PEPV: post-exposure preventive vaccination; PPV: primary preventive vaccination.

Viral DNA was sequenced from four patients to investigate genomic features potentially associated with an immune escape phenotype. Phylogenetic analysis assigned the genomes to both existing and different MPXV Clade IIb lineages (https://nextstrain.org and Figure 2). In accordance, single nucleotide variant (SNV) analysis did not show a common mutation pattern among the four viral genomes (Figure 3). Protein annotations and uniqueness of the identified SNVs in the context of publicly available MPXV sequences from the current outbreak are detailed in Supplementary Table S2. Overall, our analysis did not reveal evident nor unique genomic traits that can be linked to potential immune escape based on available knowledge on MPXV protein function.

The low viral presence in the sample of PPV Patient 3 precluded whole genome sequencing analysis at high enough precision and was excluded from the analysis.

Monkeypox virus (MPXV) genome sequencing was performed on samples from four patients (denoted in red) using a PCR-based tiling approach. Briefly, extracted DNA from anorectal or skin lesion samples was amplified using the primer sets as described [13]. Obtained amplicons were barcoded using the Oxford Nanopore rapid barcoding kit SQK-RBK004 before sequencing on a MinION flowcell (R9.4.1, Oxford Nanopore Technologies). Sequence read analysis was done essentially as described in [3] using the United States 2022 sequence ON563414.1 as a reference sequence. The 479 hMPXV genomes available at GISAID from 21 August to 21 October 2022 were used to build the phylogeny with a 2018–2019 United Kingdom reference genome (MT903344.1; denoted in green) as an external group. The phylogenetic tree was created by parsnp (default parameters) https://github.com/marbl/parsnp [14]. Branches containing no samples from our institute were collapsed for simplicity (black triangles). Our four genomes were also analysed by Nextstrain (https://clades.nextstrain.org), which assigned following lineages: B.1 to PEPV patient and PPV patient 4; B.1.3 to PPV patient 1; B.1.7 to PPV patient 2.

Numbers in the top row denote the alignment coordinates of each position. Visualisation was done by snipit (https://github.com/aineniamh/snipit). Sample collection time of reference strains was May–June 2022.

## Discussion

Our case series of five patients with severe local symptoms of mpox disease shortly after PEPV or one- or two-dose off-label PPV complements recent reports of symptomatic infections occurring shortly after vaccination [4-6]. The modified MVA-BN vaccine (also known as Imvanex, Imvamune or JYNNEOS) is a third-generation non-replicating live attenuated vaccine against variola virus (VARV), which is well-tolerated and induces good immunological responses against VARV after SC administration of two doses within a 28-day interval [7,8]. Similar results were shown for the same schedule with an ID route [9]. The rationale for its emergency use in the current mpox epidemic is due to older epidemiological studies that demonstrated cross-protection of first-generation smallpox vaccines against MPXV [10]. However, the clinical efficacy of third-generation smallpox vaccines against mpox and established correlates of protection still remain to be determined.

A few studies have been published thus far. A non-profit healthcare provider in the United States (US) recently reported 90 patients with mpox among its 7,339 vaccinees, with most infections occurring in the first 2 weeks after the first dose, although two cases were infected > 14 days after the second dose [6]. Of note, they found that eight individuals developed symptoms including some with rectal pain and proctitis more than 28 days after the first vaccine dose. Five of these infections occurred after receiving a second dose. A recent French study detected 12 (4%) PCR-confirmed mpox cases in 276 individuals who received PEPV [4]. They reported two proctitis cases that were classified as non-severe, as no hospitalisation occurred. A uniform definition of disease severity would help to facilitate comparability across studies. 

Some important limitations of these studies, including our own, are the small sample size and the problem that mild and asymptomatic cases may go undiagnosed. Nonetheless, these reports suggest that vaccinees should be encouraged to maintain other preventive measures, especially until the presumed full immunisation is reached, i.e. 2 weeks after receiving a second dose. Indeed, the four patients who were preventively vaccinated engaged in unprotected sexual intercourse shortly after vaccination with either unknown contacts or MPXV-infected individuals who were unaware of their own diagnosis and confirmed after exposure, according to information from the patients. Therefore, the benefit of continued awareness campaigns, targeted information transfer and safer sex practices should be stressed by healthcare providers.

Such campaigns might be especially important for individuals vaccinated with an off-label single-dose regimen. Recent real-world data from the US show that the average incidence of mpox was 14 times lower among individuals receiving one dose of MVA-BN compared with unvaccinated individuals, indicating that this regimen indeed offers protection on a population level [5]. Additionally, preliminary data in a preprint from Israel describe a vaccine efficacy of a single dose of 79% until day 25 in a placebo-controlled cohort with 873 vaccinated individuals [11]. However, a recent immunogenicity study showed that single-dose vaccination resulted in lower neutralising antibody levels against MPXV compared with the standard two-dose regimen [12]. Vaccinees should, therefore, be made aware of the off-label use and advised accordingly.

## Conclusion

Thus far, the control measures have had a positive impact on the mpox outbreak, as indicated by the sharp decline in new infections. The contribution of different factors like acquired herd immunity, the vaccination campaigns, changed risk behaviour and other protective measures need to be investigated. The first trial and real-world data on vaccination are promising. Nevertheless, healthcare workers, as well as those at high risk, should remain aware of the possibility of infections after vaccination, especially shortly after administration of the first dose, and be vigilant for symptoms. The importance of combining vaccination with preventive measures should be further emphasised.

## Supplementary Data

270000 Supplement
