# Supplementary material for: Severe mpox (formerly monkeypox) disease in five patients after recent vaccination with MVA-BN vaccine, Belgium, July to October 2022
Source: Euro Surveill. 2022 Dec 1;27(48):2200894. doi: 10.2807/1560-7917.ES.2022.27.48.2200894 (PMC9716643; doi:10.2807/1560-7917.ES.2022.27.48.2200894)
Supplement: Supplement [file 22-00894_BERENS-RIHA_SUPPLEMENT.pdf]

"This supplementary material is hosted by *Eurosurveillance* as supporting information alongside the article “Severe mpox (formerly monkeypox) disease in five patients after recent vaccination with MVA-BN vaccine, Belgium, July to October 2022”, on behalf of the authors, who remain responsible for the accuracy and appropriateness of the content. The same standards for ethics, copyright, attributions and permissions as for the article apply. Supplements are not edited by *Eurosurveillance* and the journal is not responsible for the maintenance of any links or email addresses provided therein."

Supplementary Table S1. Case series – clinical details

Cases	Medical history
**PEPV patient**	The first case was a 31-40-years old healthy man having sex with men (MSM) on regular pre-exposure prophylaxis (PrEP) for HIV who had unprotected oral and perceptive anal sex (“bottom”) with a monkeypox positive patient. (Table 1, Figure 1) He was traced as contact of the index patient and received a subcutaneous post-exposure vaccination (PEPV) two days later with a full dose of Imvanex. The day after, the PCR of an anal swab was already highly positive (Ct 16.15), although symptoms started eight days after exposure and six days after vaccination. After symptom onset, the viral DNA could also be detected in blood, saliva and skin lesions.The patient developed fever, fatigue and headache as well as few anal lesions in the beginning, two days later he presented with ulcerated lesions, severe rectal pain and anal secretion of blood, mucus and pus. He was prescribed antibiotics, oral and topic anti-inflammatory drugs. The severe pain could only be tolerated under opiate analgesic medication. The wounds eventually healed.
PPV patient 1	A 31-40-years old HIV-positive MSM on anti-retroviral treatment (ART, first diagnosis 2006, on treatment since 2012) was considered for a smallpox primary preventive/pre-exposure vaccination (PPV) as he was immune-compromised due to ametastasized malignancy. At presentation, he was under treatment with signal transduction inhibitors. During the night after he was vaccinated with a full subcutaneous dose of Jynneos, he had unprotected oral and insertive anal sex (“top”) with a stranger. Four days later, the patient presented at ITM with 3 lesions on the penis, followed by generalised muscle pain and fever 2-3 days later. The anal swab for MPX was positive (day 3 of symptoms, Ct 19.66). More penile lesions developed along with an inguinal lymphadenopathy and a penile oedema. Due to a bacterial super-infection of the lesions, the patient was treated with clindamycine. Some of the lesions on the foreskin ulcerated and healed with scarring and constriction. The patient was referred to an urologist for a circumcision.
PPV patients 2 and 3	A homosexual couple between 30-50 years with a high-risk profile for acquiring monkeypox infection was invited for a subcutaneous vaccination with a full dose of Jynneos. One of the men was HIV-positive, the other under continuous HIV-PrEP treatment. They were vaccinated at the same day and had 23 days later both unprotected “bottom” and “top” anal sex with two other MSM and with each other. Both had no other partners during the three weeks before onset of symptoms. 6 days later, day 29 after the first dose, they received a second dose (1/5 dose Jynneos, intradermal administration). Symptoms started two and one day(s) after the second dose in case 2 and 3, respectively. Both reported mild unspecific symptoms without fever but rectal and anal pain with mucus secretion and bleeding. They presented at an emergency department in Antwerp. Painful small anal ulcers were detected in both cases. They were empirically treated with ceftriaxone and azithromycine. Screening for gonorrhoea and chlamydia was negative, anal swab for MPX was positive. They presented 12 and 13 days after onset of symptoms at ITM, the anal swabs were still positive (Ct 25.11 and Ct 35.75, respectively). The proctitis was healed without sequelae.
PPV patient 4	A 41-50-years old healthy MSM taking regularly HIV-PrEP and practising chemsex (sex under influence of drugs) received as high-risk candidate for MPX a subcutaneous full dose Jynneos vaccine. A delayed second dose was planned. He travelled to UK where he had unprotected oral and “bottom” anal sex with a random contact 32 days after the first dose. Five days later he developed moderate rectal pain, feverish feeling, sore throat and cough. At day 8 after symptom start, he developed four moderately painful lesions in the face and subsequently contacted ITM for an appointment. He finally presented at ITM at day 16 with four big crusted, partly necrotic ulcerations in the face (Ct 26.08) and several painful anal lesions (Ct 19.80). The feverish feeling and fatigue lasted for two weeks; viral DNA was detectable in

	saliva (29.78) and blood (35.97). Topical antibiotic and local anaesthesia were prescribed. The rectal pain had stopped. The facial lesions were still not healed when this manuscript was prepared.

Supplementary Table S2. SNV contextualization

Original Name	Sample	Aligment Coordinate	Reference Coordinate	Protein Code	Annotation	NT change	AA change	# GISAID genomes with the same AA change (August 21st-October21st; n=479)	extended screen (all NCBI proteins)
22072961	PEPV_patient	69849	69848	OPG088	virion-associated glutaredoxin; Glutaredoxin-like protein	G→A	-		
22072961	PEPV_patient	85814	85813	OPG107	IMV membrane protein	C→T	-		
22072961	PEPV_patient	149016	149015	OPG174	3-b-Hydroxy-delta5-steroid dehydrogenase	G→A	-		
22072961	PEPV_patient	175265	175264	OPG204	cell surface antigen and secreted IFN-a	C→T	-		
22082796	PPV_patient_1	190705	190663	OPG016 NBT03_gp175	similar to VACV-WR N3R	G→A	R84K	17	not done
22100221	PPV_patient_2	11409	11409	OPG023	Ankyrin/Host Range (Bang-D8L); D7L; host range; ankyrin-like	C→T	-		
22100221	PPV_patient_2	25653	25652	OGP041	IFN resistance, PKR/eIF-alpha inhibitor (Cop-K3 L); C3L	C→T	E42K	19	not done
22100221	PPV_patient_2	33973	33972	OPG052	Cytoplasmic protein; similar to VACV-WR C14L and VACV-Cop F8L	C→T	R11Q	0	2
22100221	PPV_patient_2	36276	36275	OPG055	RhoA signalling inhibitor, virus release protein	C→T	D273N	0	2
22100221	PPV_patient_2	57182	57181	OPG075	virion-associated glutaredoxin; glutaredoxin 1	C→T	-		
22100221	PPV_patient_2	110294	110293	OPG129	major virion core protein p4b	G→A	-		
22100221	PPV_patient_2	144367	144366	intergenic	-	C→T	-		
22100221	PPV_patient_2	175001	175000	OPG204	cell surface antigen and secreted IFN-a	A→T	-		
22100221	PPV_casepatient_2	180653	180612	OPG209	Hypothetical protein	G→A	E31K	2	5
22100935	PPV_patient_4	26022	26021	OPG042	Phospholipase-D-like protein	C→T	G362E	0	0

Legend: The table shows the location of the SNV, amino acid changes in the involved proteins, and whether these are shared by any of the publicly available MPXV genomes from the outbreak in non-endemic countries with collection dates spanning mid August 2022 to mid October 2022 (downloaded from GISAID, https://www.gisaid.org, n = 479). To broaden this screen, the sequence of proteins with rather rare amino-acid substitutions (shared < 1%) was then used as query for the *blastp* tool (https://www.ncbi.nlm.nih.gov/) using the non-redundant database (NR), before aligning the resulting subjects using the COBALT tool (Constraint-based Multiple Alignment Tool, NCBI) to identify any other NCBI available protein sequence that shares the retrieved amino-acid substitutions.

The PPV Case 1 MPXV sequence shows a rather common (shared with 17/479 MPX sequences) R84K substitution in OPG016, a protein that contains an 'MHC class I-like antigen recognition-like superfamily'-domain (InterPro accession: 16397). The (immunologic) impact of the R84K substitution remains to be resolved.

The PPV Case 2 MPXV sequence shows non-synonymous mutations in OPG052 (R11Q) and OPG055 (D273N) that were not shared with sequences from the initial screen. The broader screen did identify two MPXV sequences that share each of these amino-acid substitutions. While OPG055 D273N was found in MPXV genome sequences from samples collected during the 2022 outbreak (OP415251.1 and OP536791.1), OPG052 R11Q could only be found in MPXV genome sequences from samples collected in the 1970's (DQ011156 .1 and KP849470 .1). A curated protein database search (https://www.ebi.ac.uk/interpro/ and https://www.uniprot.org/) did not reveal functional information for these two proteins. Of note, this MPXV genome also presented a non-synonymous mutation in OPG209 (E31K), a hypothetical protein, with low frequency in publicly available MPXV genomes (2 in the initial screen, 5 in the extended screen). Interestingly, although not unique to this MPXV genome sequence, a non-synonymous mutation was identified in protein OPG041, of which a role in IFN resistance has been shown [1]. Whether the E42K substitution results in a loss- or gain-of-function has yet to be described.

The PPV Case 4 MPXV sequence shows a non-synonymous mutation in OPG042 (G362E), a phospholipase D-like protein. Our screen did not identify other publicly available MPXV sequences that share this substitution. Of note, the gene coding for this protein was shown not to affect vaccinia virus replication nor virulence. [2,3]

Supplementary Figure S1. Severity scale for monkeypox cases.

Internal use at ITM outpatient department to categorize disease severity based on international recommendations and own experiences.

Severity	Asymptomatic	Mild	Moderate	Severe
**Clinical criteria**	• No symptoms	• Skin lesions, not infected (<25 lesions)<br>Or<br>• Nonspecific prodromic symptoms without fever or lymphadenopathy	• Disseminated skin lesions (different body regions or general rash)<br>or<br>• Any mucosal lesion (mouth, throat, anal…)<br>• 25-100 skin lesions<br>• or<br>• Ulcerative or necrotic lesion (skin or mucosal > 3 cm, not in the face)<br>or<br>• Moderate pain due to lesions (NSAID, PCT)<br>• or<br>• Prodromic symptoms with fever (>38°C sublingual)<br>or<br>• Any lymphadenopathy<br>or<br>• Viral conjunctivitis	• > 100 skin or mucosal lesions or<br>• Any complications with outpatient management:<br>- Secondary bacterial infection (with need of antibiotic treatment)<br>- Facial ulcerative or necrotic lesion (skin or mucosal > 3 cm)<br>- Abscess<br>- Proctitis<br>- Urethritis<br>- Bronchitis<br>- Pneumonia<br>- Tonsillitis<br>- Paraphimosis/penile oedema<br>- Urinary retention<br>- Cornea infection/scar<br>- Purpuric skin lesions<br>or<br>• Severe pain due to lesions/inflammation (opiate analgesic drugs)<br>**Severe-life-threatening**<br>• Hospital admission due to MPX disease (severe pneumonia, encephalitis, secondary sepsis, excruciating pain, impeding any daily activity, requiring narcotic drug…; admission solely for isolation excluded) or<br>• ICU admission due to MPX disease or<br>• Death
**Laboratory criteria**	Positive MPX-PCR	Positive MPX-PCR	Positive MPX-PCR	Positive MPX-PCR

References

1. Carroll K, Elroy-Stein O, Moss B, Jagus R. Recombinant vaccinia virus K3L gene product prevents activation of double-stranded RNA-dependent, initiation factor 2 alpha-specific protein kinase. J Biol Chem. 1993;268(17):12837-42. http://dx.doi.org/10.1016/S0021-9258(18)31463-7 PMID:8099586

2. Selvy PE, Lavieri RR, Lindsley CW, Brown HA. Phospholipase D: enzymology, functionality, and chemical modulation. Chem Rev. 2011;111(10):6064-119. http://dx.doi.org/10.1021/cr200296t PMID:21936578

3. Eckert D, Williams O, Meseda CA, Merchlinsky M. Vaccinia virus nicking-joining enzyme is encoded by K4L (VACWR035). J Virol. 2005;79(24):15084-90. http://dx.doi.org/10.1128/JVI.79.24.15084-15090.2005 PMID:16306579
